# DDX21, a Host Restriction Factor of FMDV IRES-Dependent Translation and Replication

**DOI:** 10.3390/v13091765

**Published:** 2021-09-03

**Authors:** Sahibzada Waheed Abdullah, Jin’en Wu, Yun Zhang, Manyuan Bai, Junyong Guan, Xiangtao Liu, Shiqi Sun, Huichen Guo

**Affiliations:** State Key Laboratory of Veterinary Etiological Biology, O.I.E./China National Foot-and-Mouth Disease Reference Laboratory, Lanzhou Veterinary Research Institute, Chinese Academy of Agricultural Sciences, Lanzhou 730046, China; waheed_149@yahoo.com (S.W.A.); wujinen@caas.cn (J.W.); zhangyun03@caas.cn (Y.Z.); baimanyuan@caas.cn (M.B.); guanzky@163.com (J.G.); liuxiangtao@caas.cn (X.L.)

**Keywords:** DDX21, foot-and-mouth disease virus, IRES, replication, 2B, 2C, 3C protease

## Abstract

In cells, the contributions of DEAD-box helicases (DDXs), without which cellular life is impossible, are of utmost importance. The extremely diverse roles of the nucleolar helicase DDX21, ranging from fundamental cellular processes such as cell growth, ribosome biogenesis, protein translation, protein–protein interaction, mediating and sensing transcription, and gene regulation to viral manipulation, drew our attention. We designed this project to study virus–host interactions and viral pathogenesis. A pulldown assay was used to investigate the association between foot-and-mouth disease virus (FMDV) and DDX21. Further insight into the DDX21–FMDV interaction was obtained through dual-luciferase, knockdown, overexpression, qPCR, and confocal microscopy assays. Our results highlight the antagonistic feature of DDX21 against FMDV, as it progressively inhibited FMDV internal ribosome entry site (IRES) -dependent translation through association with FMDV IRES domains 2, 3, and 4. To subvert this host helicase antagonism, FMDV degraded DDX21 through its non-structural proteins 2B, 2C, and 3C protease (3C^pro^). Our results suggest that DDX21 is degraded during 2B and 2C overexpression and FMDV infection through the caspase pathway; however, DDX21 is degraded through the lysosomal pathway during 3C^pro^ overexpression. Further investigation showed that DDX21 enhanced interferon-beta and interleukin-8 production to restrict viral replication. Together, our results demonstrate that DDX21 is a novel FMDV IRES trans-acting factor, which negatively regulates FMDV IRES-dependent translation and replication.

## 1. Introduction

**Figure 1:**
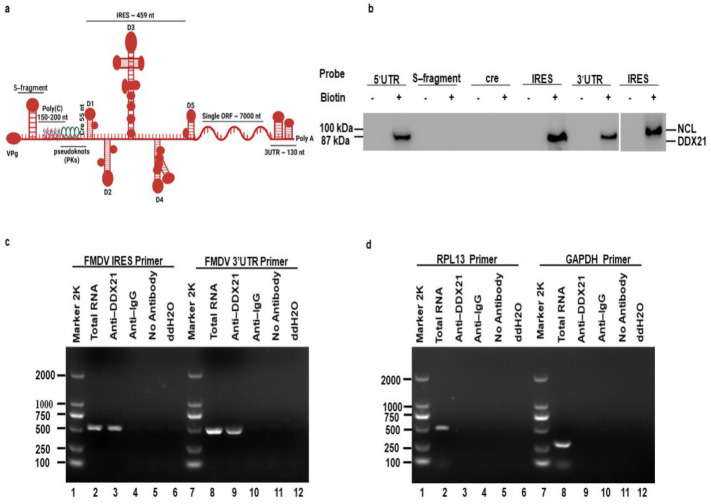
DDX21 co-precipitates with the FMDV IRES. (**a**) Schematic diagram of FMDV genome, which depict the various regions of FMDV genome. (**b**) PK-15 cells were harvested and lysed in RIPA buffer. Biotin-labeled FMDV 5′UTR, S-fragment, cre, IRES, and 3′UTR RNAs were added to the lysates and DDX21 was pulled down. Non-biotinylated RNA of each segment was used as a control. Anti-DDX21 antibodies were used for western blot analysis. (**c**, **d**) PK-15 cells were infected with FMDV at an MOI of 0.5 for 3 h. The cells were lysed in RIPA buffer, and the lysate was incubated with antibody against DDX21 for RNA immunoprecipitation. Negative controls included anti-IgG, no antibody, and ddH2O. RNA was isolated from immunoprecipitated samples, reverse transcribed, and amplified by PCR using primers directed against FMDV IRES, 3′UTR, RPL13, and GAPDH.

Protein synthesis in eukaryotes is a normal mechanism to carry on a multitude of cellular processes. Translation of mRNA is a complex process, which involves initiation, elongation, termination, and ribosome recycling [1]. Translation initiation is of two types: cap-dependent and cap-independent translation initiation [2]. During physiological conditions, cells use the cap-dependent mechanism to translate mRNA, and eukaryotic initiation factors (eIFs), the ternary complex (consisting of eIF2 (α, β, and γ subunits), GTP, and Met-tRNAi), and Met-tRNAi (a transfer RNA containing the anticodon for methionine, which initiates the translation with the help of other association factors) are recruited onto the 40S subunits to form the 43S pre-initiation complex that attaches to the 5′ region of the mRNA [1]. The cap structure protects the RNA from degradation by exonuclease cleavage and is recognized by the eIFs involved in the assembly of the ribosome [3]. The 43S pre-initiation complex scans in the 5′ to 3′ direction for translation initiation codons. A matching contact of an initiation codon with the anticodon in the Met-tRNAi switches the scanning complex to a ‘closed conformation’ that is discernible as the 48S complex. The 60S ribosomal subunit is associated with the 48S complex, which makes the 80S complex, which goes through elongation, termination, and ribosome recycling [2]. During stress, the cap-dependent translation is usually inhibited and the translation mediated by internal ribosome entry sites (IRESs) is robust and maintained [4]. Various types of IRES structures have been found in a variety of viruses [5]. Similarly, these structures were also found in cellular mRNAs, which stimulate protein translation during adverse conditions such as hypoxia, DNA damage, physiological stimuli, endoplasmic reticulum stress, and amino acid starvation [4,6,7,8,9]. The IRES-containing viruses use IRESs as primary elements for the translation of their proteins [9,10]. IRESs are classified into four types on the basis of their secondary and tertiary structures, nucleotide sequence, length, and mode of action [11]. Type I and II IRESs promote their translation initiation through a variety of eukaryotic initiation factors and IRES trans-acting factors (ITAFs) [12,13,14,15,16,17,18]. Type III IRESs only require some eukaryotic initiation factors to promote their translation initiation [19,20,21]. Type IV IRESs can initiate the translation without eukaryotic initiation factors [11,22,23,24]. Some viral IRESs hijack a variety of cellular proteins such as eukaryotic initiation factors and, more specifically, ITAFs to replicate efficiently inside the host cellular environment [5]. Foot-and-mouth disease virus (FMDV) contains a type II IRES [25], which is comprised of five domains, of which domains 2 to 5 are crucial for viral IRES-dependent translation (Figure 1a) [26,27,28,29]. Cellular ITAFs have been reported to interact with the FMDV IRES to promote or inhibit viral replication; for example, DDX3, Rab1b, Sam68, PTB, and ITAF45 [17,18,30,31] promote FMDV IRES translation, whereas Gemin5, G3BP1, hnRNP K, hnRNP L, DDX1, and DDX23 [26,32,33,34,35,36] inhibit FMDV viral translation.

So far, six RNA helicase superfamilies have been categorized based on sequence similarities and conserved motifs [37]. Among these superfamilies, superfamily 2 is the largest, containing approximately 50 RNA helicases, which have a simultaneous concomitant function in cell metabolism. The Asp-Glu-Ala-Asp (DEAD) motif possesses fundamental catalytic properties required for ATP hydrolysis [38]. DEAD-box helicases (DDXs) offer a wide array of services to the cells, playing roles in processes such as translation, transcription, RNA degradation, micro-RNA biogenesis, pre-mRNA splicing, apoptosis, gene regulation, and protein–protein interaction [38,39,40,41]. Based on these properties, it is interesting to investigate their roles in virus replication mechanisms. The effects of different helicases on different viruses vary; some facilitate, and others impede viral replication [38]. The RNA helicase DDX21 is located in the nucleolus [42]. The relationship between DDX21 and RNA/DNA viruses has been reported, showing various activities of DDX21 with different viruses. DDX21 has been shown to interfere with the pathogenic processes of human cytomegalovirus (HCMV), influenza A virus, dengue virus, human immunodeficiency virus, and Borna disease virus [43,44,45,46,47,48]. However, viruses take measures to counter-attack the host proteins to replicate and flourish in the cellular environment.

The role of DDX21 in innate immunity has also been reported. DDX21 senses DNA/RNA via pattern recognition receptors (PRRs) and induces interferon (IFN) production during poly I:C treatment, reovirus, and influenza A virus infections [49]. During influenza virus infection, DDX21 promotes the expression of S100A9, which is responsible for the induction of inflammatory and innate immune responses thorough the TLR4/MyD88 pathway against influenza A virus infection [50]. Recently, the other members of the DEAD-box family, including DDX1, DDX56, DDX3, and DDX23 [35,36,51,52], have also been reported, highlighting their immense significance in the IRES-dependent translation of FMDV.

The viral infection could result in the cleavage or degradation of host proteins to subvert the host antiviral responses. After cleavage by viruses, some proteins change their behavior and promote viral replication, as observed for hnRNP K [26]. FMDV has eight non-structural proteins: L protease (L^pro^), 2A, 2B, 2C, 3A, 3B, 3C protease (3C^pro^), and 3D polymerase (3D^pol^) [53]. The 2B protein of FMDV, also known as viroporin, induces pores in host cell membranes by disturbing the Ca^2+^ concentration, promoting cytopathy, and facilitating viral release [54]. Cyclophilin A is a host protein that is involved in the cellular response against FMDV; interaction of cyclophilin A with the FMDV 2B protein antagonizes its antiviral activity [55]. The multifunctional protein 2C, which is considered crucial for FMDV replication and has been found to interact with a wide range of host proteins, has been reported to interact with the host protein Beclin 1, indicating that the virus benefits from positive regulation of autophagy [56]. A recent study has shown that 2C interacts with the host protein NOD2 and antagonizes its antiviral activity [57]. Similarly, the crucial FMDV protease 3C^pro^ participates in the frontline defense of the virus to maintain viral integrity by inhibiting the host IFN response [58]. Our previous study confirmed that FMDV 3C^pro^ degrades DDX23 to antagonize its antiviral activity [35].

In the current study, we investigated the association between DDX21 and the FMDV IRES. During FMDV infection, DDX21 was degraded; furthermore, an increase in DDX21 mRNA levels was observed during infection. DDX21 negatively regulates FMDV IRES-dependent translation and replication. In addition, FMDV 2B, 2C, and 3C^pro^ degraded the DDX21 protein. Collectively, our results provide evidence that DDX21 plays a significant role in restricting FMDV replication. These results could be used for the development of treatment strategies against FMDV.

## 2. Materials and Methods

### 2.1. Cell Lines, Viruses, and Plasmid Constructs

PK-15 porcine kidney cells (ATCC CCL-33) and BHK-21 baby hamster kidney cells (ATCC CCL-10) were acquired from the American Type Culture Collection and cultured in 8% Dulbecco modified Eagle medium (DMEM) (Gibco Laboratories, Carlsbad, CA, USA) supplemented with 8% fetal bovine serum (FBS) (Gibco Laboratories, Carlsbad, CA, USA) and 1% penicillin/streptomycin (Gibco Laboratories, Carlsbad, CA, USA).

FMDV type O strain O/BY/CHA/2010 (GenBank accession no. JN998085.1) was obtained from the O.I.E./National Foot-and-Mouth Disease Reference Laboratory of China (Lanzhou, China) [59]. The virus was proliferated in BHK-21 cells, and TCID_50_ was used to evaluate the titer.

The coding region of the DDX21 gene was amplified with primers tagged with *Bam*HI and *Eco*RI restriction sites (5′-CGGGATCCATGCCGGGGAAACTTCGT-3′ and 5′-CGGAATTCTTACTGTCCAAACGCTTTGCTAAAACT-3′) followed by digestion and subsequent ligation into the pCMV-N-Flag vector. Similarly, the coding region of the DDX21 gene was amplified with primers tagged with *Eco*RI and *Xho*I restriction sites (forward 5′-GGAATTCATGCCGGGGAAACTTCGT-3′ and reverse 5′-CCGCTCGAGTTACTGTCCAAACGCTTT-3′) followed by digestion and ligation into the pCMV-N-HA vector. Mammalian expression plasmids for the FMDV structural proteins VP-0, VP1-2, and VP3 and non-structural proteins L^pro^, 2B, 2C, 3A, 3C^pro^, 3D^pol^, 3C-H46Y, 3C-D84N, 3C-163G, and 3C-H205R and the dual-luciferase plasmids psiCHECK-FMDV, psiCHECK-CSFV, and psiCHECK-SVV were previously synthesized by our laboratory [52,60,61]. The FMDV IRES (1–459) and truncated construct plasmids D1–2 (1–85), D3–5 (81–459), D3 (81–306), D4–5 (296–459), D4 (296–416), and D5 (407–459) were prepared in our laboratory [60]. All constructs were verified through DNA sequencing.

### 2.2. Antibodies and Reagents

Monoclonal antibodies directed against DDX21 and PTBP1 were obtained from Abcam (Cambridge, MA, USA). Monoclonal antibodies against HA and Flag tags were obtained from Proteintech (Chicago, IL, USA). Polyclonal pig antiserum against FMDV was produced in our laboratory [60]. Horseradish peroxidase, tetramethylrhodamine (TRITC) -, and fluorescein isothiocyanate (FITC)-conjugated anti-rabbit/mouse/pig antibodies and chloroquine diphosphate (CQ) were obtained from Sigma-Aldrich (St. Louis, MO, USA). The general caspase inhibitor Z-VAD(OMe)-FMK was acquired from Cell Signaling Technology (Danvers, MA, USA). The proteasomal inhibitor MG-132 was obtained from Selleck Chemicals (Houston, TX, USA). The monoclonal antibody against actin was obtained from Santa Cruz Biotechnology (Santa Cruz, CA, USA). *Bam*HI, *Eco*RI, and *Xho*I were obtained from New England Biolabs (NEB, Ipswich, MA, USA).

### 2.3. Quantitative Real-Time PCR

RNAiso Plus (Takara) was used to extract RNA from PK-15 cells, followed by reverse transcription to synthesize cDNA using 5× RT Master Mix (Takara). DDX21, FMDV, IFN-β, IL-8, and GAPDH transcript levels were quantified through quantitative real-time PCR (qRT-PCR). The primers specific for each gene were as follows: DDX21, 5′-GGACCCAAAGGGCAGCAGTT-3′ and 5′-AACGACTGGGCATCCTGCCT-3′; FMDV, 5′-CAAACCTGTGATGGCTTCGA-3′ and 5′-CCGGTACTCGTCAGGTCCA-3′; IFN-β, 5′-TGGCTGGAATGAAACCGTCA-3′ and 5′-AATGGTCATGTCTCCCCTGG-3′; IL-8, 5′-GAACTGAGAGTGATTGAGAGTGGA-3′ and 5′-GTACAACCTTCTTCTGCACCCAC-3′; and pig GAPDH, 5′-ACATGGCCTCCAAGGAGTAAGA-3′ and 5′-GATCGAGTTGGGGCTGTGACT-3′.

### 2.4. Knockdown and Overexpression

DDX21 and PTBP1 genes were knocked down using commercially synthesized small interfering (si)RNA from GenePharma (Shanghai, China). The following duplex sequences were used in PK-15 cells: to target DDX21, 5′-CCCUUUGAUUGAGAAACUUTT-3′ and 5′-AAGUUUCUCAAUCAAAGGGTT-3′; to target PTBP1, 5′-GCUGGUCAGCAACCUCAAUTT-3′ and 5′-AUUGAGGUUGCUGACCAGCTT-3′. The following negative control (NC) siRNA sequences were used: 5′-UUCUCCGAACGUGUCACGUTT-3′ and 5′-ACGUGACACGUUCGGAGAATT-3′. Duplexes were delivered via RNAi Max (Invitrogen). Samples were collected 36 h post-transfection.

PK-15 cells were transfected with mammalian expression plasmids using Lipofectamine 2000 (Invitrogen) and incubated for 24 h at 37 °C. Samples were collected after 24 h and used for Western blot, qPCR, and dual-luciferase assays.

### 2.5. TCID_50_

The supernatants of PK-15 cells, overexpressed/knocked down with DDX21, were collected, centrifuged, and 10-fold diluted. The 10-fold diluted samples were added to 96-well cell culture plates with BHK-21 cells, which were incubated for 72 h at 37 °C. TCID50 was calculated after 72 h by observing the cytopathic effect in the wells.

### 2.6. Western Blot

Cells were lysed to obtain the total protein fraction. Proteins were denatured with 1× SDS loading buffer, separated by SDS-PAGE, and transferred to PVDF membranes. Membranes were blocked for 1 h in 5% skim milk, incubated overnight with primary antibodies, washed with TBST five times, incubated with horseradish peroxidase-conjugated secondary antibodies for 90 min, and again washed five times with TBST. Finally, the membranes were incubated with enhanced chemiluminescence detection reagent (Thermo Fisher Scientific, Inc., Rockford, IL, USA) to visualize protein bands.

### 2.7. Dual-Luciferase Assay

For overexpression assays, PK-15 cells were cultured in 24-well plates. When the cells reached 80% confluency, they were co-transfected with 0.5 µg/well of pCMV-N-Flag-DDX21 and 0.5 µg/well of psiCHECK-FMDV or psiCHECK CSFV/SVV using Lipofectamine 2000. Transfected cells were incubated for 24 h, and samples were harvested with passive lysis buffer. For knockdown assays, cells were transfected with siRNA targeting DDX21 (5 µL per well) using RNAi Max and incubated for 30 h. The Firefly and Renilla luciferase activities were analyzed using the Dual-Luciferase Reporter Assay System (Promega) according to the manufacturer’s instructions.

### 2.8. In Vitro Transcription

Viral cDNAs corresponding to the 5′UTR (1–1112), the S-fragment (1–370), the cis-acting replication element (cre) (371–653), the IRES (654–1112), various truncated constructs, D1–2 (1–85), D3–5 (81–459), D3 (81–306), D4–5 (296–459), D4 (296–416), and D5 (407–459), and the 3′UTR (8112–8237) of the FMDV genome were amplified from the cDNA of FMDV strain O/BY/CHA/2010 (GenBank accession no. JN998085.1) and inserted into the pcDNA3.1 vector (Invitrogen). Next, these plasmids were linearized with BamHI, and RNA transcripts were synthesized using the RiboMAX Large Scale RNA Production System-SP6-T7 kit (Promega, Madison, WI, USA). Finally, RNA was labeled with biotin using the Pierce RNA 3′ End Desthiobiotinylation Kit per the manufacturer’s instructions (Thermo Scientific Pierce, Rockford, IL, USA).

### 2.9. RNA Pulldown Assay

Target proteins were pulled down using the Pierce Magnetic RNA-Protein Pull-Down Kit (Thermo Scientific Pierce, Rockford, IL, USA) following the manufacturer’s instructions. The experiments were performed as described in detail previously [35].

### 2.10. Nuclear Cytosol Fractionation Assay

PK-15 cells were cultured on 100 mm cell culture dishes. When a monolayer was formed, cells were infected with FMDV at a multiplicity of infection (MOI) of 0.5 for 5 h. Lysates were collected and fractionated using the Nuclear/Cytosol Fractionation Kit (BioVision, Milpitas, CA, USA) following the manufacturer’s protocol.

### 2.11. Confocal Microscopy

PK-15 cells were cultured on glass-bottom cell culture dishes (NEST, Jiangxi, China). Cells were transfected with the indicated plasmids, and after 24 h, cells were infected with type O FMDV at a MOI of 5. Immunofluorescence assays and confocal microscopy were performed as described previously [35].

### 2.12. Virus Infection

DDX21 overexpression or knockdown PK-15 cells were infected with the Chinese type O FMDV at a MOI of 0.5. The medium was changed after 1 h of infection by washing three times with 1× PBS. Cells were incubated with DMEM supplemented with 1% FBS at 37 °C, followed by sample collection at the indicated time points.

### 2.13. Proteasome, Lysosome, and Caspase Inhibitor Assays

PK-15 cells were grown to a monolayer in six-well plates and infected with FMDV or Mock infected for 1 h. After 1 h, cells were washed and incubated with DMEM supplemented with 1% FBS and the proteasome inhibitor MG-132 (10–20 µM), the caspase inhibitor Z-VAD-FMK (10–50 µM), or the lysosomal inhibitor CQ (50–100 µM). After 11 h, cells were harvested for Western blot analysis.

### 2.14. RNA Immunoprecipitation and RTPCR

PK-15 cells cultured on 100 mm cell culture dishes were transfected with the expression plasmids. At 24 h post-transfection, cells were washed twice with PBS, scratched off the plates using a cell scraper, and transferred into 1.5 mL tubes. The cells were centrifuged at 400× *g* for 5 min at 4 °C. The RIPA buffer was added to lyse the cells. The lysate was incubated on ice for 1 h (vortexing every 20 min). The lysate was centrifuged and the supernatant was used for immunoprecipitation by adding the target antibodies and incubated overnight. The protein–antibody mixture was mixed with protein G Sepharose 4 Fast Flow beads (GE Healthcare Bio-Sciences AB, Uppsala, Sweden) and incubated for 3 h at 4 °C with rotation. Beads were washed three times and eluted with RNAiso Plus for RNA extraction and RT-PCR analysis.

### 2.15. Statistical Analysis

Statistical analysis was performed using Student’s *t*-test. Experiments were performed in triplicate. *p* < 0.05 was considered to indicate statistical significance (*), *p* < 0.01 was considered to indicate strong statistical significance (**), and *p* < 0.001 was considered to indicate very strong statistical significance (***).

## 3. Results

### 3.1. DDX21 Co-Precipitates with the FMDV IRES

The FMDV 5′UTR is approximately 1300 nucleotides (nt) long and is composed of different regions. The first region is the S-fragment (350 nt), which is required for viral genome stability and replication [62]. Downstream of the S-fragment is poly(C) (150–200 nt), which is significant for the virulence of FMDV [63]. Next, the region following poly(C) is known as “pseudoknots (Pks)”, which is possibly associated with poly(C) [64]. Downstream of the Pks is cis-acting replication element (cre) (55 nt), also known as IRES domain 1, which is required for viral genome RNA replication [65]. A highly crucial IRES element (~450 nt) is located at the 3′-end of the 5′UTR, which is composed of IRES domains 2 to 5 and is important for viral IRES–dependent translation (Figure 1a) [26,27,28,29]. Host cells strongly depend on DDXs to fulfill the basic needs of cellular metabolism. Indeed, survival of cells without these helicases is impossible. Helicases provide favorable conditions for the cells to proliferate and flourish. They are involved in transcription, translation, pre-mRNA splicing, RNA degradation, gene regulation, micro-RNA biogenesis, protein–protein interaction, apoptosis, and viral replication. Based on these characteristics of helicases, we chose to investigate the role of DDX21 in FMDV replication. A pulldown assay was performed to investigate the precipitation between DDX21 and the FMDV IRES. The FMDV 5′UTR, S-fragment, cre, IRES, and 3′UTR were labeled with biotin, and PK-15 cell lysate was used to pulldown DDX21. Our Western blot results showed that, using anti-DDX21 antibodies, DDX21 was pulled down together with the FMDV 5′UTR, IRES, and 3′UTR, whereas no association was observed with the S-fragment and cre. Nucleolin was used as a positive control [60], which was pulled down together with the biotinylated FMDV IRES using anti-nucleolin antibodies (Figure 1b). To verify this interaction, we conducted an RNA co-immunoprecipitation assay. PK-15 cells were infected with FMDV for 3 h. Cells were lysed and the co-immunoprecipitation assay was performed using anti-DDX21. Finally, RNA was extracted and reverse transcribed to cDNA, followed by the amplification of desired sequences using specific primers. Primers directed against FMDV IRES and 3′UTR amplified these regions from the total RNA and immunoprecipitated samples (Figure 1c, lanes 2, 3, 8, and 9), confirming the pulldown results. In contrast, primers directed against RPL13 and GAPDH could amplify these genes in total RNA samples (Figure 1d, lanes 2 and 8), but not in immunoprecipitated samples (Figure 1d, lanes 3 and 9). In addition, no amplification was observed in negative control (NC) immunoprecipitated samples using anti-IgG, no antibody, or ddH2O (Figure 1c,d, lanes 4–6 and 10–12).

### 3.2. DDX21 Pulldown with the FMDV IRES Regions

**Figure 2:**
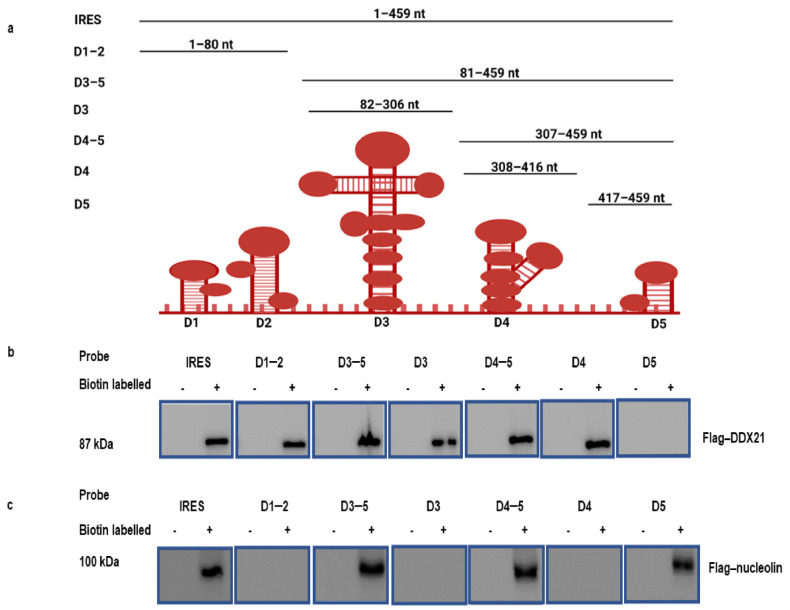
Regions of the FMDV IRES precipitate with the host protein DDX21. (**a**) Schematic diagram of FMDV IRES domains. (**b**) PK-15 cells in 100 mm cell culture dishes were transfected with 10 µg of Flag-DDX21. The lysate was collected in RIPA buffer and mixed with the in vitro synthesized biotinylated full-length FMDV IRES and domains D1–2, D3–5, D3, D4–5, D4, and D5. Following pulldown, beads were eluted with elution buffer, and 1× SDS loading buffer was added for Western blot analysis. (**c**) PK-15 cell in 100 mm cell culture dishes were transfected with 10 µg of Flag-nucleolin. The lysate was collected in RIPA buffer and mixed with the in vitro synthesized biotinylated full-length FMDV IRES and domains D1–2, D3–5, D3, D4–5, D4, and D5. Following pulldown, beads were eluted with elution buffer, and 1× SDS loading buffer was added for Western blot analysis.

Because DDX21 co-precipitates with the FMDV IRES, we investigated which regions of the FMDV IRES precipitate with DDX21. The FMDV IRES structure in living cells has been recently resolved by SHAPE, chemical, and enzymatic analyses [66,67]. According to the M-FOLD-predicted FMDV IRES secondary structures, FMDV IRES (1–459) and truncated construct plasmids D1–2 (1–80), D3–5 (81–459), D3 (82–306), D4–5 (307–459), D4 (308–416), and D5 (417–459) (Figure 2a) were prepared in our laboratory [60]. These truncated FMDV IRES domains were used to pulldown Flag-DDX21 through a RNA pulldown assay. PK-15 cells were then transfected with Flag-DDX21 and incubated for 24 h. The cell lysate was collected in RIPA buffer and mixed with biotinylated FMDV IRES and its truncated regions. Our Western blot results indicate that Flag-DDX21 was pulled down with the FMDV IRES and its domains D1–2, D3–5, D3, D4–5, and D4; however, no pulldown was observed with D_5_ (Figure 2b). Flag-nucleolin was used as a positive control, which showed its pull down with FMDV IRES and domains D3–5, D4–5, and D5 (Figure 2c) [60]. These results indicate which FMDV IRES domains took part in the association with DDX21.

### 3.3. DDX21 Negatively Regulates FMDV Replication

**Figure 3:**
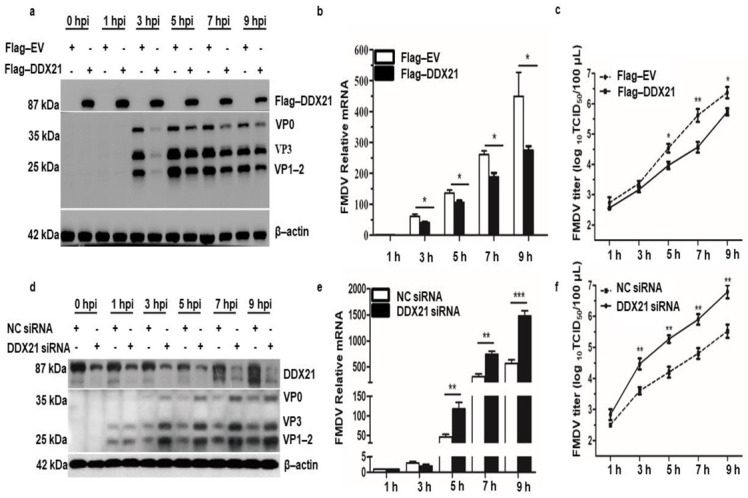
DDX21 inhibits FMDV replication. (**a**) PK-15 cells in 12-well plates were transfected with Flag-DDX21 plasmid (1.5 µg) and incubated for 24 h at 37 °C. Infection with type O FMDV was performed at a MOI of 0.5 and cells were incubated for an additional 24 h at 37 °C. Samples were collected at 0, 1, 3, 5, 7, and 9 hpi. Cell lysates were analyzed by Western blotting. (**b**) PK-15 cells in 12-well plates were transfected with Flag-DDX21 plasmid (1.5 µg) and incubated for 24 h at 37 °C. Infection with type O FMDV was performed at a MOI of 0.5 and cells were incubated for 1 h at 37 °C. Samples were collected at 0, 1, 3, 5, 7, and 9 hpi using RNAiso Plus for RNA extraction and qRT-PCR analysis. (**c**) PK-15 cells in 12-well plates were transfected with Flag-DDX21 plasmid (1.5 µg) and incubated for 24 h at 37 °C. Infection with type O FMDV was performed at a MOI of 0.5 and cells were incubated for 1 h at 37 °C. Cells were washed with 1× PBS and incubated in DMEM supplemented with 1% FBS. Samples were collected at 0, 1, 3, 5, 7, and 9 hpi. Cellular supernatants were collected, centrifuged, and stored at −80 °C for TCID50 analysis. (**d**) DDX21 was knocked down using siRNA directed against DDX21. Cells were incubated for 36 h, followed by FMDV type O infection. Cell lysates were analyzed by Western blotting. (**e**) DDX21 was knocked down using siRNA directed against DDX21. Cells were incubated for 36 h, followed by FMDV type O infection. RNAiso Plus was added to collect samples for RNA extraction and qRT-PCR analysis. (**f**) DDX21 was knocked down using siRNA directed against DDX21. Cells were incubated for 36 h, followed by FMDV type O infection. Cellular supernatants were collected, centrifuged, and used for TCID_50_ analysis. The data are presented as the mean and SD of three separate experiments (* *p* < 0.05, ** *p* < 0.01, *** *p* < 0.001).

We have shown that FMDV infection induced DDX21 degradation. Therefore, we investigated the role of DDX21 in FMDV replication. Flag-DDX21 and Flag−EV (empty vector) plasmids were transfected into PK−15 cells, which were infected with type O FMDV at a MOI of 0.5. DDX21 overexpression significantly decreased FMDV protein at 3, 5, 7, and 9 hpi (Figure 3a). A gradual decrease in the Flag-tagged DDX21 protein level was observed during FMDV infection (Figure S1). Likewise, a decline in FMDV mRNA levels was observed in DDX21 overexpression cells (Figure 3b). TCID_50_ showed a similar trend; the viral titer reduced compared with the Flag-EV-infected cells (Figure 3c). The cell viability assay was performed to check whether Flag-DDX21 transfection affects the cell viability and proliferation. The cell viability assay revealed that overexpression of Flag-DDX21 did not affect cellular viability and proliferation (Figure S2).

We further confirmed our findings using siRNA directed against DDX21 or NC. PK-15 cells were infected with FMDV type O at a MOI of 0.5 at 36 h post-transfection. The Western blot results show an increase in FMDV protein expression at 3, 5, 7, and 9 hpi (Figure 3d). Similarly, qRT-PCR results show that the FMDV mRNA levels were increased in DDX21 knockdown cells compared with NC samples (Figure 3e). The supernatants were collected at indicated time points and TCID50 was evaluated. The viral titer showed a similar pattern, indicating an increased amount of virus in knockdown cells (Figure 3f). These results suggest that DDX21 counter-attacked FMDV and restricted viral replication.

### 3.4. DDX21 Negatively Regulates FMDV, CSFV, and SVV IRES-Dependent Translation

**Figure 4:**
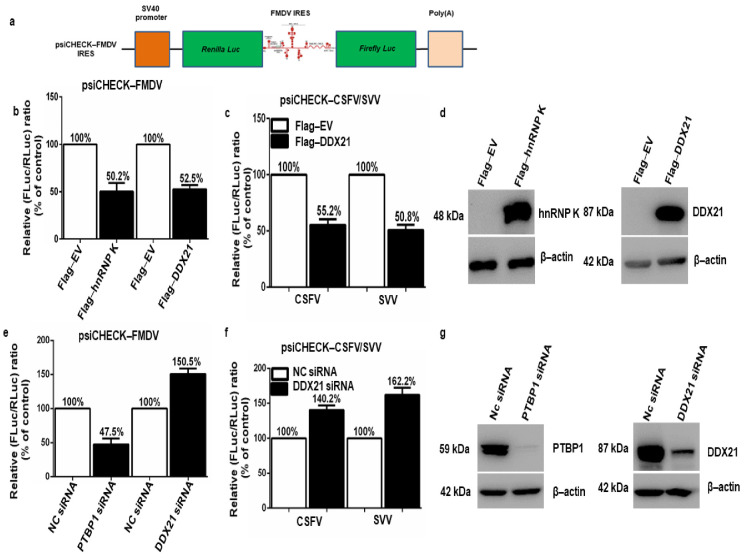
DDX21 inhibits FMDV, classical swine fever virus (CSFV), and Seneca Valley virus (SVV) IRES-dependent translation. (**a**) Schematic diagram of the bicistronic luciferase construct. (**b**) PK-15 cells in 24-well plates were co-transfected with psiCHECK-FMDV and Flag-DDX21, Flag-hnRNP K (positive control), or Flag-EV (negative control). Samples were harvested 24 h post-transfection using passive lysis buffer or 1× SDS loading buffer for Western blot analyses. The dual-luciferase assay was performed using the Dual-Luciferase Reporter Assay System. (**c**) PK-15 cells in 24-well plates were co-transfected with psiCHECK-CSFV or psiCHECK-SVV and Flag-DDX21 or Flag-EV. (**d**) PK-15 cells were transfected with Flag-DDX21 or Flag-EV and Flag-hnRNP K or Flag-EV. Samples were collected 24 h post-transfection for Western blot analyses. (**e**) PK-15 cells in 24-well plates were knocked down with siRNA-DDX21, siRNA-PTBP1 (positive control), or siNC (negative control). At 30 h post-knockdown, cells were transfected with psiCHECK-FMDV. After 24 h, samples were collected using passive lysis buffer for luciferase activity analysis. (**f**) PK-15 cells in 24-well plates were knocked down using siRNA-DDX21 or siNC. After 30 h, cells were transfected with psiCHECK-CSFV or psiCHECK-SVV. After an additional 24 h, samples were collected using passive lysis buffer for luciferase activity analysis. (**g**) PK-15 cells were knocked down with siRNA-DDX21, siRNA-PTBP1, or siNC. Samples were collected at 36 h post-transfection for Western blot analysis.

The inhibitory effect of DDX21 on FMDV protein, mRNA levels, and viral titer led us to explore FMDV (type II IRES), classical swine fever virus (CSFV) (type III IRES) [20], and Seneca Valley virus (SVV) (type III IRES) [21] IRES-dependent translation. Figure 4a depicts the bicistronic FMDV IRES construct (psiCHECK-FMDV). To investigate the role of DDX21 in different viral IRES translation initiation mechanisms, PK-15 cells were co-transfected with Flag-DDX21 or Flag-EV and psiCHECK-FMDV, psiCHECK-CSFV, or psiCHECK-SVV and incubated for 24 h. The hnRNP K, which negatively regulates FMDV IRES-dependent translation, was used as a positive control [26]. The dual-luciferase assay results indicate that Flag-hnRNP K and Flag-DDX21 decreased FMDV IRES-dependent translation by 50.2% and 52.5%, respectively (Figure 4b). In addition, CSFV and SVV IRES activity was decreased by 55.2% and 50.8%, respectively, in DDX21 overexpression samples (Figure 4c). The transfection efficiency was confirmed through Western blot analyses, which showed the protein expression of Flag-DDX21 and Flag-hnRNP K in overexpression samples (Figure 4d). Next, DDX21 and PTBP1 were knocked down using siRNA targeting DDX21 and PTBP1, respectively. After 30 h, the cells were transfected with psiCHECK-FMDV, psiCHECK-CSFV, or psiCHECK-SVV. SiRNA targeting PTBP1, which positively regulates FMDV IRES-dependent translation, was used as a positive control [68]. The dual-luciferase assay results show that DDX21 knockdown increased FMDV, CSFV, and SVV IRES activity by 150.5%, 140.2%, and 162.2%, respectively (Figure 4e,f). In the positive control (PTBP1 knockdown), FMDV IRES activity decreased by 52.5% (Figure 4e). The knockdown efficiencies of siRNAs targeting DDX21 and PTBP1 were assessed through Western blot analyses, which showed a significant decrease in DDX21 and PTBP1 protein expression (Figure 4g). Tables S1 and S2 show the absolute values of firefly luciferase and *Renilla* luciferase. The fact that DDX21 suppressed FMDV, CSFV, and SVV IRES-dependent translation suggested that DDX21 had broad-spectrum activity.

### 3.5. DDX21 Translocates to the Cytoplasm during FMDV Infection

**Figure 5:**
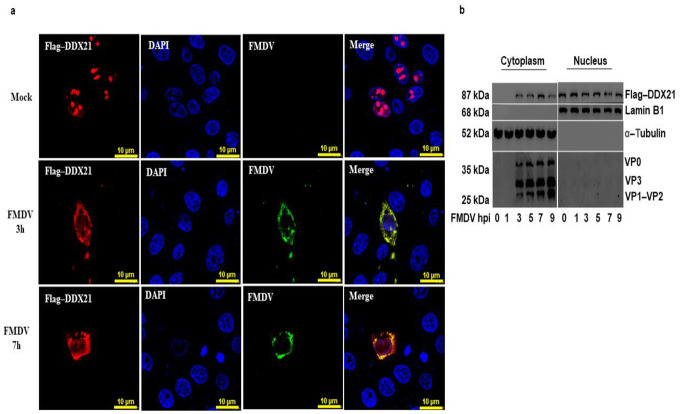
DDX21 is translocated into the cytoplasm of FMDV-infected cells. (**a**) PK-15 cells were cultured in glass-bottom cell culture dishes and transfected with Flag-DDX21, followed by mock or FMDV infection. Cells were fixed with 4% paraformaldehyde at 3 and 7 hpi. An indirect immunofluorescencent antibody test was performed using primary anti-Flag antibodies and secondary TRITC-conjugated antibodies (red); polyclonal pig antiserum was prepared in our laboratory [60], which was used to detect the viral proteins with secondary FITC-conjugated antibodies (green). Nuclei were stained blue with DAPI; the merged signal appeared yellow. (**b**) PK-15 cells on 100 mm dishes and transfected with 2 µg of Flag-DDX21. Cells were FMDV-infected, and samples were collected at 0, 1, 3, 5, 7, and 9 hpi. Samples were processed using the nuclear/cytosol fractionation assay. The nuclear and cytosol fractions were analyzed through Western blot assay.

We have shown that DDX21 was pulled down with FMDV IRES domains. Considering that FMDV replication occurs in the cytoplasm, we hypothesized that DDX21 was translocated to the cytoplasm to interact with FMDV. To investigate this, PK-15 cells were cultured in glass-bottom cell culture dishes and transfected with Flag-DDX21. Cells were infected with FMDV type O or mock-infected and fixed with 4% paraformaldehyde at 3 and 7 hpi. An indirect immunofluorescent antibody test was performed with anti-DDX21 to localize DDX21 and determine its translocation during viral infection. In mock-infected cells, DDX21 (red) resided in the nucleolus; however, upon FMDV (green) infection, DDX21 was translocated into the cytoplasm, where FMDV replication occurs (Figure 5a). These results were further confirmed through a nuclear/cytosol fractionation assay. PK-15 cells were transfected with Flag-DDX21. Cells were infected with FMDV type O. Cellular samples were collected at the indicated time points and the nuclear/cytosol fractionation assay was performed. Flag-DDX21 was mainly observed in the cytoplasm of FMDV-infected cells at 3, 5, 7, and 9 hpi; however, it was not observed in the cytoplasmic fraction at 0 and 1 hpi (Figure 5b). These results suggest that DDX21 translocated into the cytoplasm of the FMDV-infected cell.

### 3.6. FMDV Infection Causes Degradation of the DDX21 Protein

**Figure 6:**
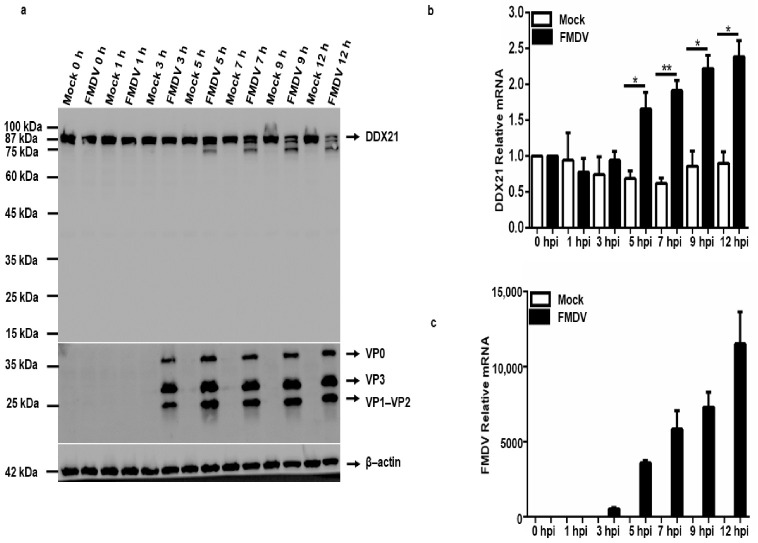
FMDV degrades DDX21 during viral infection. (**a**) PK-15 cells in 12-well plates were mock-infected or infected with type O FMDV at a MOI of 0.5, and samples were harvested at 0, 1, 3, 5, 7, 9, and 12 hpi. Collected samples were analyzed by Western blotting. (**b**,**c**) PK-15 cells in 12-well plates were infected with type O FMDV, and cells were harvested using RNAiso Plus reagent for RNA collection. RNA was extracted, and qRT-PCR was performed. The data are presented as the mean and SD of three separate experiments (* *p* < 0.05, ** *p* < 0.01).

To evaluate DDX21 protein and mRNA expression during FMDV infection, PK-15 cells were infected with type O FMDV at a MOI of 0.5 and harvested at the indicated time points. Our Western blot results show that DDX21 was degraded at 5, 7, 9, and 12 h post-infection (hpi) (Figure 6a). We did not observe DDX21 degradation at 0, 1, and 3 hpi (Figure 6a). Next, we infected PK-15 cells with type O FMDV at a MOI of 0.5 and collected samples at the indicated timepoints using RNAiso Plus for RNA extraction. Interestingly, the qPCR results show a significant increase in DDX21 mRNA transcript levels at 5, 7, 9, and 12 hpi (Figure 6b) compared with mock-infected cells. Figure 3c shows the FMDV mRNA levels over time. No viral RNA was detected in mock-infected cells. These results suggest that FMDV infection triggered DDX21 mRNA expression, whereas the DDX21 protein was degraded during viral infection.

### 3.7. FMDV 2B, 2C, and 3C^pro^ Decrease DDX21 Protein Levels, and 3C^pro^ Catalytic Triad Active Site Residues Are Required for DDX21 Degradation

**Figure 7:**
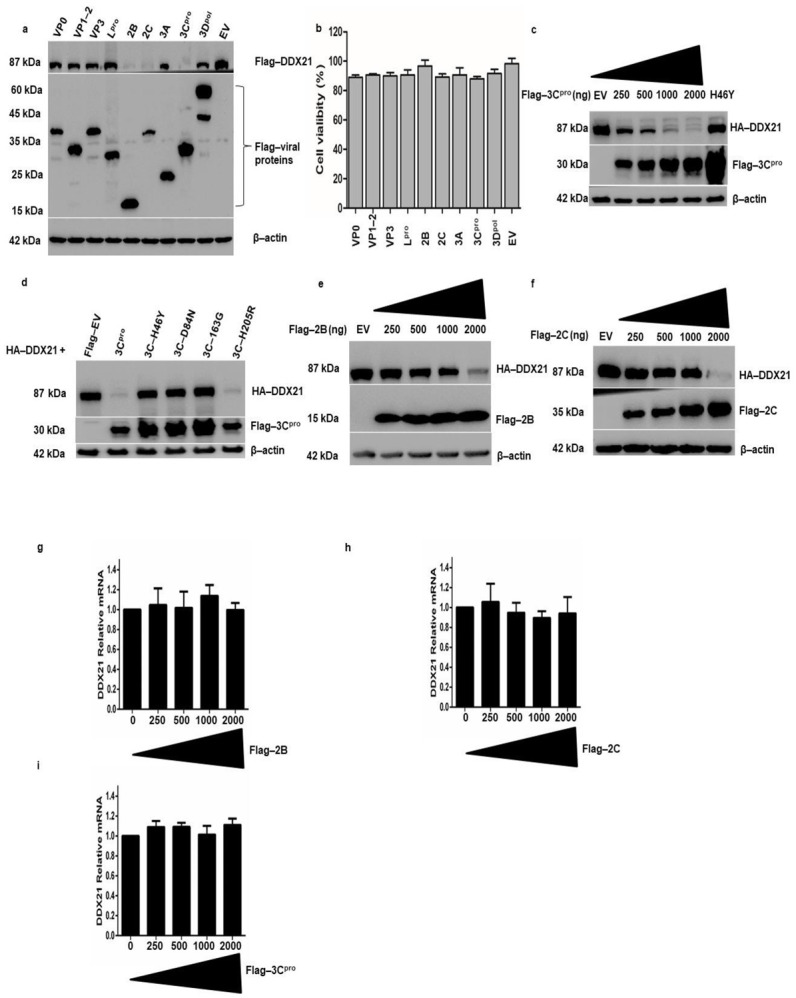
DDX21 is degraded by FMDV 2B, 2C, and 3C^pro^. (**a**) PK-15 cells in six-well plates were co-transfected with Flag-DDX21 and VP0, VP1-2, VP3, L^pro^, 2B, 2C, 3A, 3C^pro^, 3Dpol, or Flag-EV. Cells were harvested after 24 h and 1× SDS loading buffer was added. The samples were analyzed by Western blot. (**b**) After the cells were grown to 80% confluence in 96 well plates, they were transfected with Flag-VP0, VP1-2, VP3, L^pro^, 2B, 2C, 3A, 3C^pro^, and 3D^pol^ or an empty vector for 24 h. For the MTS assay, 10 μL of CellTiter 96^®^ AQueous One Solution Cell Proliferation Assay reagent (Promega, WI, USA) was directly added to the cells, which were then incubated for 4 h. The absorbance at 490 nm was recorded. (**c**) PK-15 cells were co-transfected with HA-DDX21 (2 µg) and Flag-3C^pro^ (250, 500, 1000, or 2000 ng) or Flag-EV (2 µg). Cell lysates were collected in 1× SDS loading buffer and analyzed by Western blotting. (**d**) PK-15 cells on six-well plates were co-transfected with HA-DDX21 (2 µg) and Flag-3C^pro^, H46Y, D84N, 163G, H205R, or Flag-EV (2 µg). Samples were collected at 24 h post-transfection and analyzed by Western blotting. (**e**) PK-15 cells on six-well plates were co-transfected with HA-DDX21 (2 µg) and Flag-2B (250, 500, 1000, or 2000 ng) or Flag-EV (2 µg). Cell lysates were collected 24 h post-transfection in 1× SDS loading buffer and analyzed by Western blotting. (**f**) PK-15 cells were co-transfected with HA-DDX21 (2 µg) and Flag-3C^pro^ (250, 500, 1000, or 2000 ng) or Flag-EV (2 µg). Cell lysates were collected 24 h post-transfection in 1× SDS loading buffer and analyzed by Western blotting. (**g**–**i**) PK-15 cells were cultured on a six-well plate. At 80% confluence, cells were transfected with an increasing concentration of Flag-2B, Flag-2C, and Flag-3C^pro^ (0, 250, 500, 1000, and 2000 ng). Twenty-four hours post transfection; RNA was extracted and the level of DDX21 mRNA was determined by qRT-PCR.

The degradation of DDX21 by FMDV infection prompted us to investigate which viral proteins were responsible for this degradation. PK-15 cells were transfected with Flag-DDX21 and Flag-VP1-2, VP3, L^pro^, 2B, 2C, 3A, 3C^pro^, 3pol, or EV. Samples were collected after 24 h and analyzed through Western blotting. FMDV 2B, 2C, and 3C^pro^ significantly decreased DDX21 protein levels, whereas VP0, VP1-2, VP3, L^pro^, 3A, and 3Dpol did not change the DDX21 protein levels (Figure 7a). The cell viability assay showed that overexpression of FMDV structural and non-structural proteins did not affect cellular viability and proliferation (Figure 7b). Next, we transfected PK-15 cells with HA-DDX21 and Flag-3C^pro^ with increasing concentrations (250, 500, 1000, or 2000 ng). With increasing Flag-3C^pro^ concentration, DDX21 was more quickly degraded (Figure 7c), which indicated that DDX21 degradation was dose-dependent. PK-15 cells were co-transfected with Flag-DDX21 and Flag-3C^pro^ or its mutants H46Y, D84N, and C163G [60], in which the catalytic residues were mutated, and the constitutively catalytically active Flag-3C^pro^ H205R [60]. Western blot analysis showed that wild-type 3C^pro^ and H205R decreased DDX21 protein levels; however, upon transfection with 3C^pro^ mutants in which the catalytic residues were mutated, i.e., H46Y, D84N, and C163G, DDX21 protein levels were not reduced (Figure 7d). To investigate the effects of increasing concentrations of Flag-2B or 2C on DDX21 protein levels, we co-transfected PK-15 cells with HA-DDX21 and Flag-2B or 2C. We observed a significant decrease in DDX21 protein levels after transfection with 25 to 2000 ng of Flag-2B- or 2C-encoding plasmids (Figure 7e,f). The significant decrease in DDX21 in the presence of 2B or 2C was only observed with the 2000 ng concentration in contrast to 3C^pro^, in which inhibition started at the 250 ng concentration. These results confirm that FMDV 2B, 2C, and 3C^pro^ were involved in the degradation of DDX21. To evaluate whether the decrease in DDX21 was the result of a specific decrease in mRNA transcripts, PK-15 cells were transfected with an increasing concentration of Flag-2B, Flag-2C, and Flag-3C^pro^ (0, 250, 500, 1000, and 2000 ng) and mRNA was extracted 24 h post-transfection. The qRT-PCR results show that no significant change was observed in the DDX21 mRNA during the overexpression of Flag-2B, Flag-2C, and Flag-3C^pro^ (Figure 7g–i). These results indicate that DDX21 was degraded by Flag-2B, Flag-2C, and Flag-3C^pro^ only at the proteomic level, which could be due to lysosomal, proteasomal, or caspase pathway dependence.

### 3.8. DDX21 Does Not Interact with FMDV 2B, 2C, and 3C^pro^

**Figure 8:**
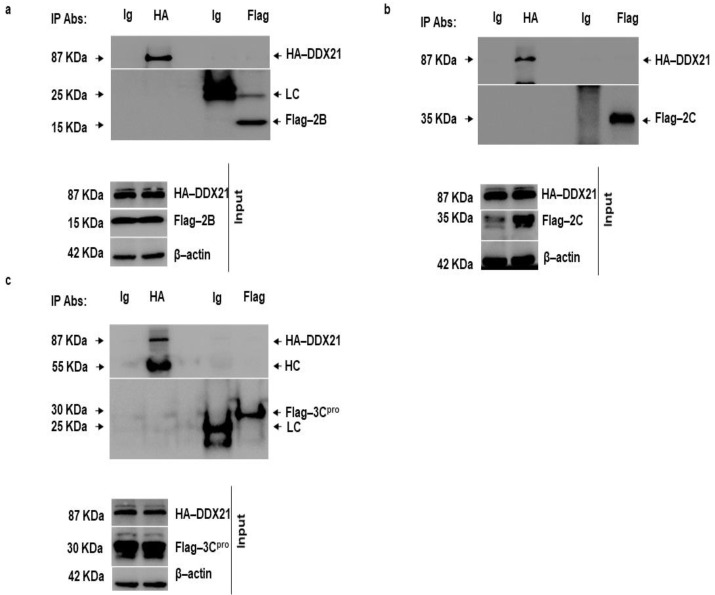
DDX21 does not interact with FMDV 2B, 2C, and 3C^pro^. (**a**) PK-15 cells in 100 mm cell culture dishes were co-transfected with HA-DDX21 (8 µg) and Flag-2B (6 µg) and incubated for 24 h. Protein lysates were collected in RIPA buffer. Forward immunoprecipitation was performed with anti-HA and reverse immunoprecipitation was performed with anti-Flag. The immunocomplexes were analyzed by SDS-PAGE and Western blotting. (**b**) PK-15 cells in 100 mm cell culture dishes were co-transfected with HA-DDX21 (8 µg) and Flag-2C (6 µg) and incubated for 24 h. Protein lysates were collected in RIPA buffer. Forward immunoprecipitation was performed with anti-HA and reverse immunoprecipitation was performed with anti-Flag. The immunocomplexes were analyzed by SDS-PAGE and Western blotting. (**c**) PK-15 cells in 100 mm cell culture dishes were co-transfected with HA-DDX21 (8 µg) and Flag-3C^pro^ (6 µg) and incubated for 24 h. Protein lysates were collected in RIPA buffer. Forward immunoprecipitation was performed with anti-HA and reverse immunoprecipitation was performed with anti-Flag. The immunocomplexes were analyzed by SDS-PAGE and Western blotting.

FMDV 2B, 2C, and 3C^pro^ decreased DDX21 protein levels; therefore, we investigated whether DDX21 directly interacted with these FMDV non-structural proteins. PK-15 cells were co-transfected with HA-DDX21 and Flag-2B, 2C, or 3C^pro^. After 24 h, the cell lysates were obtained in RIPA buffer, and a co-immunoprecipitation assay was performed. Forward immunoprecipitation was accomplished with anti-HA and reverse immunoprecipitation was accomplished with anti-Flag-antibodies. IgG was used as a negative control. Forward immunoprecipitation showed that DDX21 did not precipitate 2B, 2C, or 3C^pro^. Similarly, reverse immunoprecipitation showed that 2B, 2C, or 3C^pro^ did not precipitate DDX21 (Figure 8a–c).

### 3.9. Lysosomal and Caspase Pathway-Dependent Degradation of DDX21

**Figure 9:**
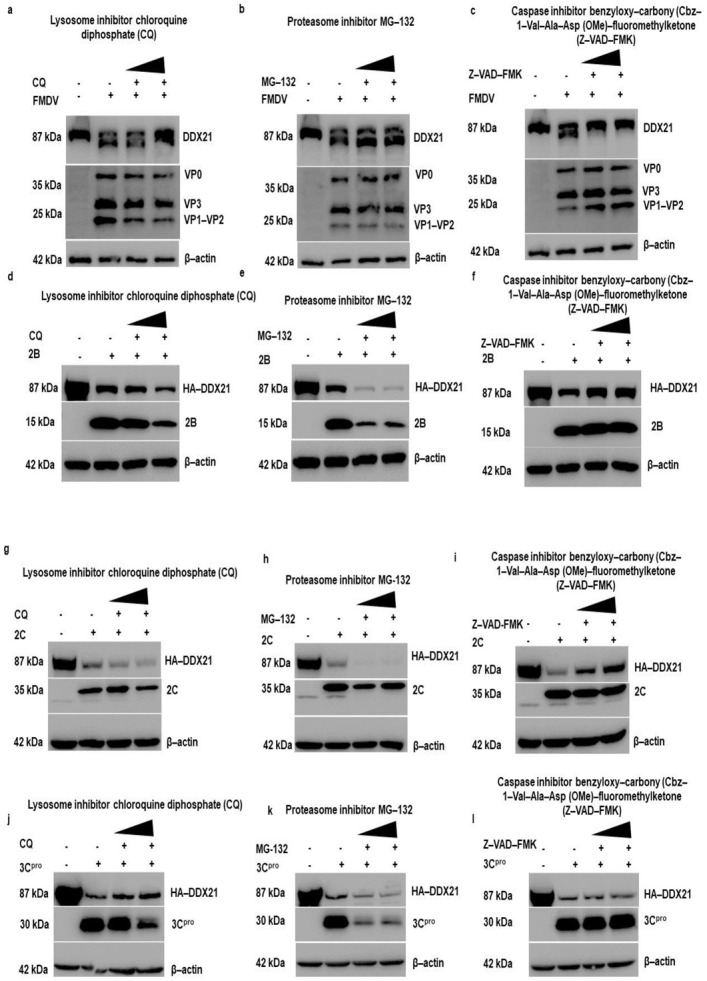
DDX21 is degraded through the caspase pathway during FMDV infection. (**a**) PK-15 cells in six-well plates were infected with FMDV type O at a MOI of 0.5 and incubated for 1 h. Cells were washed with 1× PBS three times, and CQ was added at 50 to 100 µM to inhibit the lysosome pathway. After 11 h of incubation, samples were harvested in 1× SDS loading buffer, and Western blot analyses were performed. (**b**) PK-15 cells in six-well plates were infected with FMDV type O at a MOI of 0.5 and incubated for 1 h. Next, cells were washed with 1× PBS three times, and MG-132 was added at 10 to 20 µM to inhibit proteasome pathways. After 11 h of incubation, samples were harvested in 1× SDS loading buffer, and Western blot analyses were performed. (**c**) PK-15 cells in six-well plates were infected with FMDV type O at a MOI of 0.5 and incubated for 1 h. Next, cells were washed with 1× PBS three times, and Z-VAD-FMK was added at 10 to 50 µM for the inhibition of caspase pathways. After 11 h of incubation, samples were harvested in 1× SDS loading buffer, and Western blot analyses were performed. (**d**–**l**) PK-15 cells in six-well plates were co-transfected with HA-DDX21 (2 µg) and Flag-2B, 2C, 3C^pro^, or Flag-EV (2 µg). At 6 h post-transfection, cells were washed with 1× PBS, CQ was added at 50 to 100 µM, MG-132 was added at 10 to 20 µM, and Z-VAD-FMK was added at 10 to 50 µM, and cells were incubated for an additional 18 h and collected in 1× SDS loading buffer. Samples were analyzed by SDS-PAGE and Western blot.

To explore which protein degradation pathway is responsible for the FMDV-dependent degradation of DDX21, PK-15 cells were infected with FMDV, and after 1 h of infection, the inhibitors CQ, MG-132, and Z-VAD(OMe)-FMK were added to inhibit the lysosomal, proteasomal, and caspase pathways, respectively [69]. Our Western blot results show that DDX21 protein levels were entirely restored by the use of Z-VAD-FMK (Figure 9c); However, the DDX21 protein levels were not restored when CQ or MG-132 was used (Figure 9a,b). These results suggest that upon FMDV infection, DDX21 was degraded through the caspase pathway. Next, we investigated which pathways were involved in the degradation of DDX21 by 2B, 2C, and 3C^pro^. Our results indicate that 2B and 2C degraded DDX21 via the caspase pathway (Figure 9f,i), and not via the lysosome and proteasome pathways (Figure 9d,e,g,h); and 3C^pro^ degraded DDX21 via the lysosomal pathway (Figure 9j), and not via the proteasome or caspase pathways (Figure 9k,l). These results suggest that DDX21 was degraded through lysosomal and caspase pathways.

### 3.10. DDX21 Positively Regulates IFN-β and IL-8 Production

**Figure 10:**
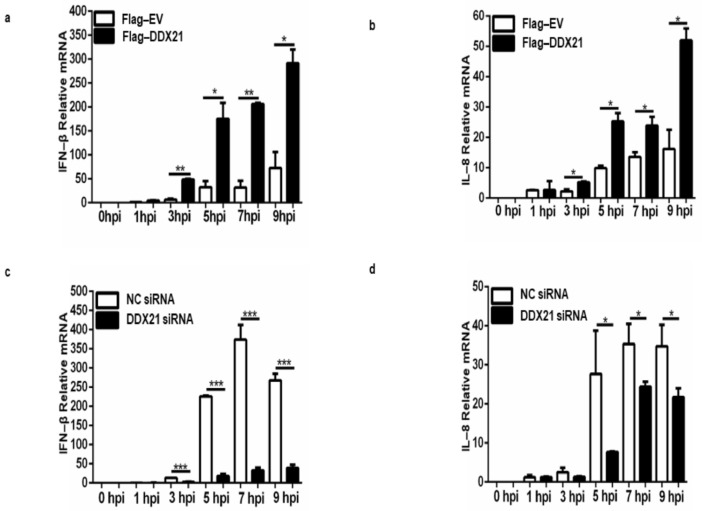
DDX21 induces IFN-β and IL-8 production during FMDV infection. (**a,b**) PK-15 cells in 12-well plates overexpressing Flag-DDX21 or Flag-EV were infected with FMDV type O at a MOI of 0.5. Samples were harvested with RNAiso Plus at 0, 1, 3, 5, 7, and 9 hpi. RNA was extracted and qRT-PCR analysis was performed. (**c,d**) PK-15 cells in 12-well plates were knocked down using siRNA-DDX21 or siNC, incubated for 36 h, and infected with FMDV type O. Samples were collected at 0, 1, 3, 5, 7, and 9 hpi using RNAiso Plus. RNA was extracted, and qRT-PCR was performed. The data reflect the means of three separate trials and error bars indicate standard deviations (SD) (* *p* < 0.05, ** *p* < 0.01, *** *p* < 0.001).

Our results show that DDX21 inhibits viral replication; therefore, we decided to evaluate IFN-β and IL-8 production during viral infection. PK-15 cells were transfected with Flag-DDX21 or Flag-EV, incubated for 24 h, and infected with FMDV type O. Samples were collected at the indicated timepoints. During DDX21 overexpression, IFN-β and IL-8 production was significantly increased compared with Flag-EV samples (Figure 10a,b). Next, we confirmed these results through DDX21 knockdown. DDX21 was knocked down, and at 36 h post-transfection cells were infected with FMDV type O. Samples were collected using RNAiso Plus for RNA extraction. Our qPCR results show that during FMDV infection, IFN-β and IL-8 mRNA levels were significantly decreased in the DDX21 knockdown cells compared with the scrambled siRNA negative control (NC) samples (Figure 10c,d). These results suggest that DDX21 was an anti-FMDV agent, which inhibited viral replication via an innate immune response.

## 4. Discussion

Viruses must multiply with limited resources, even though a multitude of processes are required for replication; hence, they depend upon the host to proliferate and flourish [70]. The hostile conditions of the host cell pose obstacles to replication.

Nevertheless, FMDV utilizes the indispensable weapon IRES to hijack a variety of host proteins known as ITAFs to promote translation of the viral mRNA [11]. Studies have reported new ITAFs that modulate FMDV IRES-mediated translation and replication; however, there is still a scarcity of information to explain the viral IRES-mediated translation mechanism. Here, we report a novel FMDV ITAF, the RNA helicase DDX21, that co-precipitates with the FMDV IRES and whose overexpression negatively regulates viral IRES-dependent translation and replication. In addition, DDX21 translocated into the cytoplasm during FMDV infection; however, DDX21 was degraded through FMDV 2B, 2C, and 3C^pro^, antagonizing its antiviral activity.

DDX21 is involved in a multitude of cellular processes, including cell growth, protein translation, ribosome biogenesis, RNA-protein and protein-protein interactions, rDNA transcription, mediating and sensing transcription during nucleotide stress, and regulation of a variety of genes [71,72,73,74,75]. The influence of DDX21 is not limited to cellular processes, but extends to the regulation of viral pathogenesis and replication. Various reports have shown that DDX21 responds differently to a variety of viruses; DDX21 negatively regulates influenza A virus replication by interacting with PBP1 and inhibiting viral polymerase [43]. Similarly, DDX21 inhibits dengue virus infection by translocating to the cytoplasm and stimulating the innate immune response [45]. In contrast, DDX21 promotes HCMV replication and protein expression, and the mRNA levels of DDX21 are increased in virus-infected cells; DDX21 knockdown decreases viral growth in human fibroblasts. HCMV is a DNA virus, which could explain why DDX21 promotes viral replication [47]. The current study revealed that DDX21 was pulled down with FMDV IRES domains 1, 3, and 4, which resulted in a reduction in FMDV mRNA levels, protein expression, and viral titer. To our knowledge, this is the first report that describes the co-precipitation of DDX21 and the FMDV IRES domains. Furthermore, our dual-luciferase assay showed that DDX21 not only suppressed FMDV IRES-dependent translation, but was also involved in the suppression of CSFV and SVV IRES-dependent translation. Based on our observations, we propose that DDX21 acts as a broad-spectrum antagonist against RNA viruses.

Translocation of host proteins during viral infection has been reported. The translocation of host proteins may promote or inhibit translation of viral proteins. For example, during Sindbis virus infection, the host protein XRN1 is translocated to the cytoplasmic viral replication factories and promotes viral replication [76]. The host protein FBP2, a negative regulator of EV71 virus replication, is translocated to the cytoplasm during EV71 infection [77]. Based on the aforementioned reports, we decided to evaluate the DDX21 translocation status, and we found that DDX21 was translocated from the nucleolus to the cytoplasm of FMDV-infected cells. One possible reason for this translocation could be the association of DDX21 with FMDV IRES and the subsequent suppression of FMDV IRES-dependent translation.

According to the hostile conditions inside host cells, viruses adapt themselves and strive to combat the life-threatening host proteins [78,79]. In many circumstances, they succeed in hunting down host proteins by taking proper measures such as cleaving or degrading host proteins. As a result of this cleavage or degradation, viruses can subvert the host antiviral activity [80]. FMDV uses its two proteases, L^pro^ and 3C^pro^, to cleave its polyprotein as well as a wide range of host proteins [81]. Surprisingly, some proteins upregulate viral replication after cleavage by a viral protease. For example, FMDV 3C^pro^ cleaves hnRNP K into a C-terminal and an N-terminal cleavage product; the C-terminus of hnRNP K upregulates FMDV replication activity [26]. FMDV cleaves G3BP1 and G3BP2 through its L protease to antagonize and escape from the host antiviral response [82]. The third crucial non-structural FMDV protein, 2B, also known as viroporin, degrades RIG-I to evade the host antiviral response [69]. We evaluated the protein and mRNA levels of DDX21 during FMDV infection. DDX21 was degraded during FMDV infection, and an increase in DDX21 mRNA levels was observed. The increase in mRNA levels could be due to the normal cellular process where DDX21 increases its mRNA transcript to suppress FMDV replication at the transcriptional level. Similar phenomena were previously reported for DDX23, DDX1, and RIG-I, whose mRNA levels are higher in FMDV-infected cells compared with mock-infected cells [35,69,83].

Further investigation showed that DDX21 protein expression was reduced by FMDV 2B, 2C, and 3C^pro^. FMDV 3C^pro^ cleaves NEMO, which works as an adapter molecule in the MDA5/RIG-I pathway, to inhibit IFN production [84]. 3C^pro^ was reported to inhibit host protein translation during FMDV infection [85]. The present study indicates that FMDV 3C^pro^ results in DDX21 degradation, which is partly due to the enzymatic activity of 3C^pro^. Degradation of DDX21 was not observed in catalytically inactive 3C^pro^ mutants such as H46Y, D84N, and 163G, although the constitutively catalytically active mutant H205R showed degradation of DDX21 similar to wild-type 3C^pro^ levels. A previous report has shown that FMDV 2B reduced the RIG-I protein expression [69]. The highly conserved FMDV protein 2C was reported to induce apoptosis [86]. DDX21 is believed to perform a function in the cell cycle and maintain cellular growth; therefore, 2C may degrade DDX21 to induce apoptosis and facilitate the release of viral particles [71,87]. Therefore, we transfected cells with 2B, 2C, and 3C^pro^ at increasing concentrations, which resulted in a consistent decrease in DDX21 protein levels; however, no interaction was detected between DDX21 and the FMDV non-structural proteins 2B, 2C, and 3C^pro^. A previous study has shown that the dose-dependent increase in 3C^pro^ and L^pro^ decreased the MDA5 mRNA level and protein expression [88]. Contrastingly, no change in RIG-I mRNA level was observed during the expression of FMDV 2B [69]. In the current study, the mRNA transcript of DDX21 was evaluated during the Flag-2B, 2C, and 3C^pro^ transfection, which showed no significant change in the DDX21 mRNA level. No change in the DDX21 mRNA level during the expression of FMDV 2B, 2C, and 3C^pro^ suggested that the decrease in DDX21 only occurred at the proteomic level where FMDV and its non-structural proteins 2B, 2C, and 3C^pro^ could possibly degrade DDX21 via lysosome, proteasome, or caspase pathways. A recent report has shown that FMDV L^pro^ and 3C^pro^-dependent degradation of MDA5 is independent of lysosomal, proteasomal, or caspase pathways [88]. Similarly, FMDV 2B-dependent degradation of RIG-I was also independent of these pathways [69]. In the current study, we investigated the involvement of the lysosome, proteasome, and caspase pathways in FMDV-, 2B-, 2C-, and 3C^pro^-dependent degradation of DDX21, which showed that FMDV, 2B, and 2C use the caspase pathway to degrade DDX21. In contrast, 3C^pro^ utilizes the lysosomal pathway to degrade DDX21 protein. The different protein degradation pathways used by 2B, 2C, and 3C^pro^ suggest that host proteins may be degraded due to the unique behavior of FMDV proteins.

It has been previously reported that DDX21 induces type I interferon responses against RNAs. Knockdown of DDX21 significantly reduced IFN-β production following treatment of cells with poly I:C and during reovirus and influenza A viral infection [49]. In our study, we observed a similar trend of IFN-β production following DDX21 knockdown. Hence, it is suggested that DDX21 activates IFN-β production against a wide range of viruses. The pro-inflammatory cytokine IL-8 is secreted by cells during transmissible gastroenteritis virus infection; however, knockdown of DDX1 decreased its secretion [36]. In the current study, we showed that during FMDV infection, DDX21 knockdown decreased IL-8 production and DDX21 overexpression increased IL-8 production, suggesting that DDX21 overexpression promoted IL-8 secretion during FMDV infection to combat the invading pathogen.

In conclusion, our study revealed that DDX21 co-precipitates with FMDV IRES and that overexpression of DDX21 restricted viral IRES-dependent translation and replication. DDX21 translocated into the cytoplasm of FMDV-infected cells. FMDV and its non-structural proteins 2B, 2C, and 3C^pro^ reduced DDX21 expression. FMDV-, 2B-, and 2C-dependent degradation of DDX21 were due to the caspase pathway, whereas 3C^pro^ used the lysosomal pathway for the degradation of DDX21. In addition, the overexpression of DDX21 increased IFN-β and IL-8 production in FMDV-infected cells to subvert the viral infection. Thus, DDX21 is an antagonist and a novel FMDV ITAF. These properties of DDX21 could be utilized to develop new FMDV treatment strategies.

## Data Availability

All data are included in the manuscript.

## Supplementary Materials

The following are available online at https://www.mdpi.com/article/10.3390/v13091765/s1, Figure S1: A gradual decrease in Flag-tagged DDX21 protein levels was observed during FMDV infection. Figure S2: Flag-DDX21 overexpression has no effect on the cellular viability and proliferation. Table S1: Absolute values of firefly luciferase and *Renilla* luciferase. Table S2: Absolute values of firefly luciferase and *Renilla* luciferase.
