# Supplementary material for: DDX21, a Host Restriction Factor of FMDV IRES-Dependent Translation and Replication
Source: Viruses. 2021 Sep 3;13(9):1765. doi: 10.3390/v13091765 (PMC8473184; doi:10.3390/v13091765)
Supplement: Supplementary file 1 [file viruses-13-01765-s001.zip › viruses-1231098-supplementary.pdf]

viruses MDPI

Supplementary File: DDX21, a Host Restriction Factor of FMDV IRES–Dependent Translation and Replication

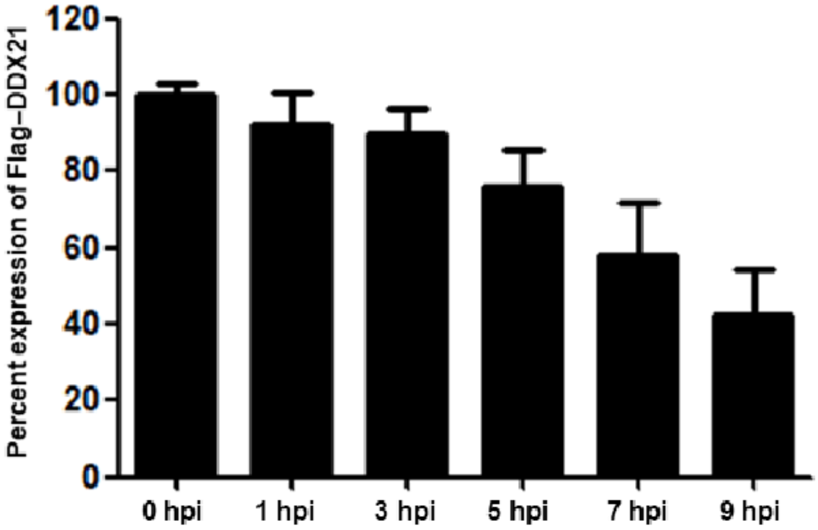

Figure S1. A gradual decrease in Flag-tagged DDX21 protein levels was observed during FMDV infection. The bands of Flag-DDX21 were analyzed through ImaheJ software.

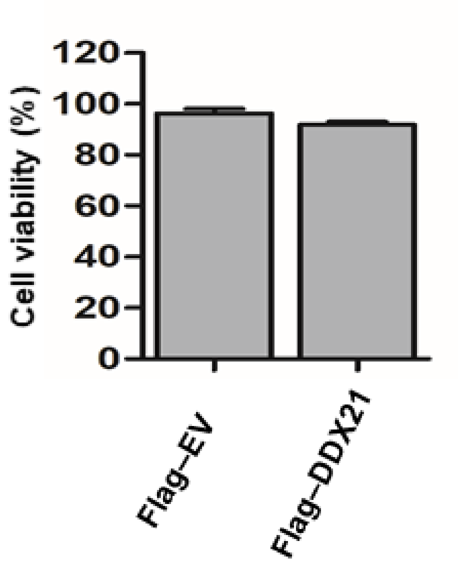

Figure S2. Flag-DDX21 overexpression has no effect on the cellular viability and proliferation. After the cells were grown to 80% confluence in 96 well plates, they were transfected with Flag-DDX21 or Flag-empty vector (EV) for 24 h, for the MTS assay, 10 μL of CellTiter 96® Aqueous One Solution Cell Proliferation Assay reagent (Promega, WI, USA) was directly added to the cells, which were then incubated for 4 h. The absorbance at 490 nm was recorded.

Table S1. Absolute values of firefly luciferase and *Renilla* luciferase.

		FLuc/RLuc								
Flag-EV-psiCHECK-FMDV	1	75664505/1384898371	75475467/1683595578	73856026/1904110299	71978430/2208726267	68659590/2264361193	73063398/2268785168	76390727/1945045606	74752235/524500008	77603744/1126498457
	2	64089594/1690507610	57906841/1906319131	69322033/2026695426	61078103/1803331817	101666886/1871243852	78770494/1876644078	84320073/1927864037	79344011/1830642831	93240770/1821485894
	3	64951014/1713229487	58685159/1931941700	70253781/1948375956	61899045/1948210148	103033376/1941634979	79829237/1901867788	85453408/1953776188	80410463/1930648246	94494006/1845968231
Flag-DDX21- psiCHECK-FMDV	1	35922176/1226919424	31134176/1724775040	39229240/1575977984	41812992/1812197760	50161388/1943348736	49142032/1849107968	51098132/1832979968	46583220/1737728000	46066184/1616751104
	2	34991166/1823918425	42162184/1835676039	44291252/1866076985	33982193/1812197659	41171427/1943224730	43041033/1850211872	46098131/1872939997	41383121/1877528001	46099183/1876741413
	3	38162171/1925929131	27994181/1925785353	37229235/1974959594	43822997/1813567950	50161377/1836388706	49942041/1948128968	51018122/1842776723	46583231/1936627182	46066183/1915455214
Flag-EV-psiCHECK-CSFV	1	33980844/891622841	29960793/1171094272	38363284/2053138911	39374216/1920679307	38910116/1970609989	48840411/1973237460	39019205/1994575378	40690578/1679519013	48093442/1661791651
	2	35630399/1982986771	31415201/1811915856	38929580/2007963011	34805586/1878417960	40798956/1927250000	49915305/1929819659	43505341/1950688071	42665849/1642563996	50428075/1625226695
	3	35218010/2011531665	31051599/1816099778	38479006/2293902512	34402744/1638234043	40326746/1694019902	49337582/1696955487	43001807/2228471463	42172032/1876469665	49844417/1856663484
Flag-DDX21- psiCHECK-CSFV	1	17535746/1714833423	16507651/1657132275	22041172/1700465839	20707078/1640368447	26957848/1752416543	29466516/1680917079	33270094/1671890900	30585574/1800095557	30866770/1695918046
	2	18525634/1942353655	15517763/1888534695	11051263/1911807943	32295087/2040917728	27865799/1908828973	28576287/1747843699	24381211/1747997206	21771133/1833286545	21676284/1736118196
	3	15126554/1848621092	21016843/1786418222	23041184/1833132580	19107068/1966502583	21979648/1968398794	21124714/1970583520	23211173/1901406317	20591582/1920719591	20562441/1927308010
Flag-EV-psiCHECK-SVV	1	60251702/1099250733	50589030/1543624220	61491606/1652885998	48425308/1642215874	42905226/2061449003	67612491/1897474379	92522050/1768459753	77962490/1822150230	75005890/1826908933
	2	61154403/1768722619	51346963/1776094549	62412883/2106479236	49150824/1655207648	43548039/2077757383	68625472/1912485535	93908230/1782450258	79130536/1836565487	76129641/1841361837
	3	59857666/2079775065	50258186/1829449644	61089461/1958942896	48108615/1946297037	42624633/2443157536	67170317/1730832928	91916971/1836923086	77452628/1641561370	74515364/1647201219
Flag-DDX21- psiCHECK-SVV	1	33849400/1798084029	26682300/1635187358	30739880/1736658732	31630080/1625468986	30815944/1764038088	32878168/1721351154	31103724/1662718381	31002388/1638702044	30924856/1733679036
	2	32858114/	35573631/	31859880/	32585475/	31816862/	37878239/	37922641/	49403467/	41633744/

		1914574794	1881830381	1853577426	1872179714	1880766039	1838376491	1909169604	1885320581	1721601077
	3	33385012/ 1897065572	26633211/ 1939874968	31218649/ 1909033534	30711291/ 1830635495	31535931/ 2104439163	37616243/ 1821025117	34013482/ 1859573462	30102639/ 1846422095	32724643/ 1913228521
Flag-EV-psiCHECK-FMDV	1	78900216/ 1672555758	80310787/ 1864006324	78790991/ 1631107479	69946050/ 1622225489	65379791/ 1692476521	80127610/ 1678210171	70359274/ 1746568820	62853098/ 1689358850	80668750/ 1718478506
	2	66380579/ 1845106652	78051617/ 1874658183	96099172/ 1874303269	75360641/ 1854267370	67303652/ 1834905761	72852899/ 1830132969	75864639/ 1861327414	76660170/ 1873631807	80975866/ 1904840117
	3	67268298/ 1834143717	79095416/ 1828870021	97384323/ 2003911631	76368452/ 1886561255	68203715/ 1963599337	73827174/ 1947954696	76879190/ 1915307285	77685360/ 1852570179	82058771/ 1884503126
Flag-hnRNP K- psiCHECK-FMDV	1	31667367/ 1722795377	29541968/ 1713762150	28944996/ 1692606875	29556262/ 1715656649	29423177/ 1723785995	47877105/ 1725607593	29129027/ 1708315960	29054632/ 1647810362	29460367/ 1664978051
	2	36689912/ 1938870839	36630785/ 1832105476	37512390/ 1809489329	36648176/ 1931239148	36715960/ 1842821514	36427287/ 1941877251	36358087/ 1826283201	36726976/ 1858707756	37220606/ 1779952605
	3	45991928/ 1902993955	43466495/ 1893120006	44963990/ 1879924789	48136105/ 1895190826	36249105/ 1983508610	47354187/ 1906067904	49842714/ 1916953879	49751061/ 1920319882	52576923/ 1819937609

Table S2. Absolute values of firefly luciferase and *Renilla* luciferase.

FLuc/RLuc										
NC siRNA- psiCHECK-FMDV	1	1837486/ 435178935	2347016/ 430973875	2513300/ 468562361	3190463/ 460878069	3131666/ 469053546	3530563/ 472253427	4090690/ 445448753	4056419/ 401468769	4181434/ 402646761
	2	3608585/ 383559443	3351606/ 394173450	3744692/ 380352292	2918033/ 421524154	3828256/ 429001536	3229092/ 431928182	3259390/ 407412332	3228046/ 333447754	4306386/ 368265159
	3	3577322/ 479719745	3555708/ 526677280	3761434/ 579701441	3947215/ 504994499	3874471/ 515109131	4367983/ 453867997	4408967/ 551105478	4366568/ 483653809	4521235/ 498151211
DDX21 siRNA-psiCHECK-FMDV	1	3841689/ 400972288	4678687/ 415580768	5117673/ 429870816	5089794/ 406330400	5002308/ 435266688	4833481/ 424546272	5677759/ 483553088	6474849/ 501793728	6027104/ 494606688
	2	5752747/ 411883377	4767468/ 362868679	5238555/ 387871925	5969172/ 391239299	4411216/ 457155579	5124573/ 342257381	5766868/ 490422177	5235740/ 291214639	4127104/ 449879957
	3	6752578/ 411783399	5767798/ 606629657	4928784/ 420261725	5278683/ 612139218	5113417/ 642357577	5722372/ 411455361	5580648/ 471232325	5544960/ 533545362	6116216/ 482415353
NC siRNA-psiCHECK-CSFV	1	16358690/ 443487704	17433283/ 448089123	18087419/ 447905042	17100116/ 439475643	17638701/ 438891699	16161953/ 442605900	18567708/ 439339224	18968750/ 439609006	17925786/ 447341891
	2	14565285/ 379517063	15522070/ 393991501	16104493/ 364614112	15225428/ 394643376	15704968/ 403964947	14390116/ 405362953	16532128/ 365509722	16889204/ 380381715	15960580/ 461325203

	3	11194462/ 460910223	15157820/ 521121285	12377453/ 520907201	11701829/ 467216189	15298389/ 510424811	11059832/ 514744375	16902521/ 510945276	16208559/ 511259030	15494845/ 575111941
DDX21 siRNA- psiCHECK-CSFV	1	24791157/ 404130090	26791242/ 427544234	28093051/ 441271454	13732733/ 422619850	28008905/ 442344527	27012953/ 429717846	28989738/ 461792787	29168210/ 485661900	27715527/ 477157314
	2	27185640/ 324479498	28572132/ 459102540	19474568/ 354300616	9519758/ 455148711	23416236/ 355162196	18725826/ 460847752	20096166/ 370777370	25219886/ 389942085	19212862/ 383113680
	3	11394551/ 493107706	10863221/ 503697378	19895479/ 502874714	19928847/ 514890818	18625225/ 512636392	19616747/ 523253092	24187277/ 510056314	23128732/ 521181993	21134548/ 511162590
NC siRNA- psiCHECK-SVV	1	9182110/ 401999378	11195333/ 405406055	5552953/ 402160874	10628588/ 423897740	11040322/ 422773164	11174311/ 424804531	11191569/ 409515004	11424499/ 529910078	10977758/ 565827552
	2	12858001/ 415575907	11735664/ 417870006	9941904/ 415684660	11280273/ 365938303	11611109/ 365180999	11718772/ 366548947	14472639/ 356252789	14659803/ 461472418	11560838/ 385864129
	3	15950491/ 514445879	11159220/ 517806408	12333040/ 396712999	10594302/ 536047595	11004708/ 534938253	11138265/ 536942102	11155467/ 415756726	11387646/ 581677742	14341346/ 558162566
DDX21 siRNA- psiCHECK-SVV	1	18521706/ 429180492	19012240/ 433899395	8014929/ 444806045	20096414/ 437010274	19816878/ 445440653	19243660/ 446884656	19563338/ 459341881	19920796/ 455507347	17068280/ 440620113
	2	18432617/ 359687151	22121339/ 422386703	22042317/ 372782598	21025302/ 424993865	18105763/ 373314450	20356472/ 374524638	19678289/ 414337146	17617432/ 381751134	17144356/ 428019195
	3	13810023/ 494105463	19352685/ 498775506	17998972/ 509569228	19373923/ 501854174	19821535/ 527539451	20657482/ 511626316	17241845/ 523954558	21835924/ 520159727	19953821/ 505426637
NC siRNA- psiCHECK-FMDV	1	2769801/ 407597233	3020481/ 408875149	3264047/ 426487199	2966405/ 391834705	3030408/ 395503299	3099535/ 393716476	3589051/ 510924225	3547801/ 516015867	3668570/ 532644532
	2	3762443/ 350488865	3505793/ 351587732	3729591/ 366732161	3699598/ 364526535	3027933/ 367681123	2847959/ 407532220	4271707/ 439338733	3259841/ 443716986	3370807/ 458015811
	3	4037484/ 485502519	4682087/ 525245148	4883199/ 508002981	2449346/ 466727252	4690284/ 471097036	2559271/ 468968691	5151553/ 513027557	3804639/ 519092380	4123166/ 634450485
PTBP1 siRNA- psiCHECK-FMDV	1	1360361/ 327665544	1541785/ 389712857	1692906/ 414848061	2059401/ 505883038	2046024/ 506842514	2133857/ 514183791	1868380/ 448576632	1693310/ 445884770	1919658/ 441789342
	2	1364401/ 370334158	1421133/ 412920837	1511292/ 388618804	1310578/ 471878294	1305368/ 493313730	1514985/ 352914230	2103689/ 307884222	1965292/ 451474887	2141297/ 303225711
	3	2146266/ 545876658	1782583/ 516773786	1888566/ 511577690	1444282/ 511801622	1434900/ 482146955	1496498/ 489574121	1310316/ 484450129	1187538/ 512352144	1346278/ 538834171
